# Individual differences in vocal size exaggeration

**DOI:** 10.1038/s41598-022-05170-6

**Published:** 2022-02-16

**Authors:** Michel Belyk, Sheena Waters, Elise Kanber, Marc E Miquel, Carolyn McGettigan

**Affiliations:** 1 Department of Speech Hearing and Phonetic Sciences, University College London, London, UK; 2 Department of Psychology, Edge Hill University, Ormskirk, UK; 3 Wolfson Institute of Preventative Medicine, Queen Mary University of London, London, UK; 4 Clinical Physics, Barts Health NHS Trust, London, UK; 5 Digital Environment Research Institute, Queen Mary University of London, London, UK

**Keywords:** Human behaviour, Social evolution

## Abstract

The human voice carries socially relevant information such as how authoritative, dominant, and attractive the speaker sounds. However, some speakers may be able to manipulate listeners by modulating the shape and size of their vocal tract to exaggerate certain characteristics of their voice. We analysed the veridical size of speakers’ vocal tracts using real-time magnetic resonance imaging as they volitionally modulated their voice to sound larger or smaller, corresponding changes to the size implied by the acoustics of their voice, and their influence over the perceptions of listeners. Individual differences in this ability were marked, spanning from nearly incapable to nearly perfect vocal modulation, and was consistent across modalities of measurement. Further research is needed to determine whether speakers who are effective at vocal size exaggeration are better able to manipulate their social environment, and whether this variation is an inherited quality of the individual, or the result of life experiences such as vocal training.

## Introduction

The voice is a common carrier for a wide range of communicative signals. The most obvious of these signals are speech, song, and the expression of emotion, but the voice also carries socially relevant information about the person to whom it belongs^1^. Listeners attribute a range of traits to speakers based on even very brief exposure to a person’s voice^2,3^. Among these percepts is an estimate of the speaker’s body size: taller people tend to have longer vocal tracts, and this can be detected from the spacing of formant frequencies in their voice.

Speakers can volitionally modulate the acoustics of their voice^4–6^ to modulate their apparent size^7^. In social interactions, vocal size exaggeration contributes to the impression of higher-order social traits such as authority^8^, social dominance^9–11^, and masculinity/femininity^4,5,8,12–14^. Favourable evaluations along these traits can impact social and professional outcomes across the lifespan^15–18^.

Vocal size is conveyed by the properties of the vocal tract as an acoustical resonator. The space between the larynx and the lips is highly labile and changes shape to form the various phonemes of speech^19,20^. Articulatory movements shape resonant cavities of varying sizes in the vocal tract, which in turn selectively filter or amplify particular frequency bands in the acoustical signal that listeners perceive as a voice^21^. These resonances are called formants. While dynamic changes to the shape of vocal tract resonant cavities, and their corresponding formants, are the primary carriers of the phonemic content of speech, the overall size of the vocal tract also varies between individuals. Shorter speakers have shorter vocal tracts that amplify high-frequency components in their voice, while taller speakers have longer vocal tracts that amplify low-frequency components of the voice^22,23^. However, the larynx can be raised by a system of muscles which attach the larynx to the tongue and jaw (effectively shortening the vocal tract), or lowered by a system of muscles which attach the larynx to the sternum and clavicles (effectively lengthening the vocal tract^24^).

Magnetic resonance imaging provides a non-invasive means of measuring the shape and size of the vocal tract^25^. This technology can be used to quantify differences between the vocal tracts of speakers^23,26–28^ and dynamic changes to the shape of the vocal tract within speakers^29,30^. Singers for instance, have particular expertise in using vocal tract modulation in service of switching between modal and falsetto vocal registers^31,32^.

While the muscles involved in vocal size exaggeration are under voluntary control, not all speakers control them equally well^33–35^, suggesting the possibility that some speakers may be more effective vocal size modulators than others. Recent work from our group using vocal tract MRI and functional MRI of brain activation during the imitation of large and small voice targets reported group differences in larynx lowering and raising behaviours related to singing expertise^36^, but neither group nor individual differences in this behaviour were analysed in relation to corresponding voice acoustics or listeners’ perception of talker size. Given that size modulation may influence social interactions, there is a need for an account of how individual differences in this skill manifest across vocal tract modulations, speech acoustics, and ultimately their influence over the perceptions of listeners.

**Figure 1:**
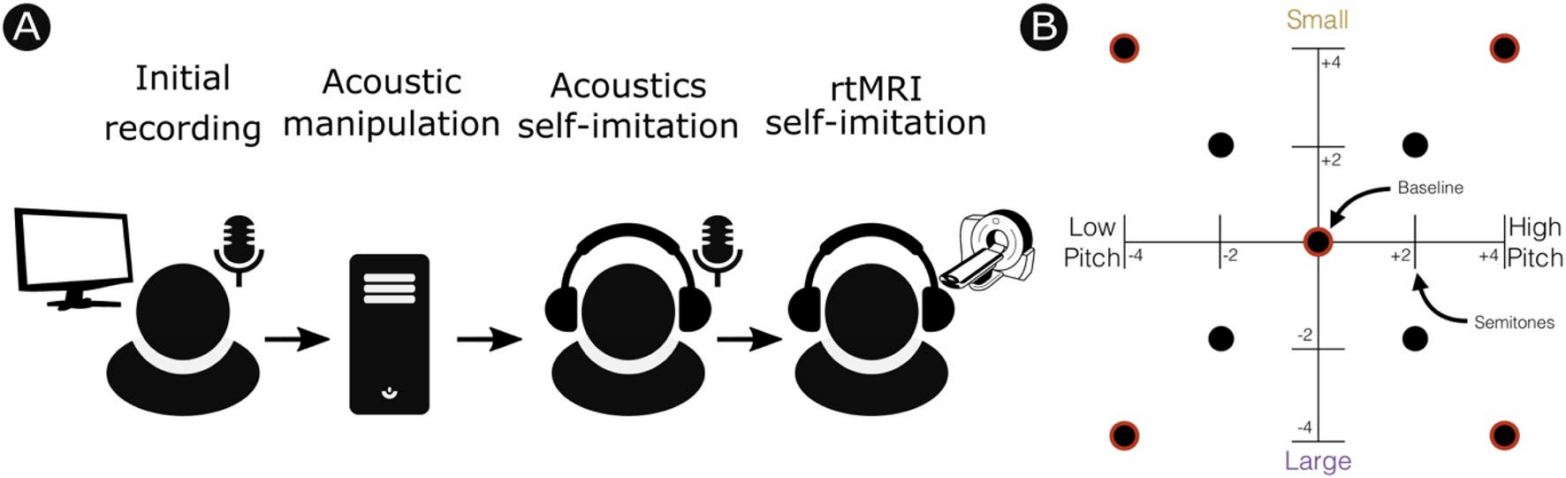
(**A**) Overview of methodology for experiment 1. Participants were first recorded producing the carrier words BEAD and BARD. These recordings were acoustically manipulated to have higher or lower vocal pitch, and narrower or wider formant spacing to imply a smaller or larger body size. Participants then imitated the manipulated recordings separately in a sound attenuated booth to produce audio recordings suitable for acoustical analyses, and again in an MRI scanner for vocal tract imaging. (**B**) Summary of acoustic manipulations. The self-imitation for acoustics session included all conditions shown (± 2 or 4 semitones). The self-imitation for rtMRI session included only the conditions outlines in red (± 4 semitones).

We assessed individual differences in the ability to exaggerate vocal size using multimodal measurements of vocal tract modulation. Novel analyses of real-time Magnetic Resonance Imaging (rtMRI) from a previous voice imitation study^36^ were used to quantify the degree to which speakers were able to modulate the shape of their vocal tract (see Fig. 1), and in turn to modulate speech acoustics. Finally, a new perception experiment tested whether speakers who produce larger vocal tract movements have greater influence over listeners’ judgements of talker height.

## Results

### Experiment 1: real-time MRI

Functional principal components analysis (fPCA) revealed that 5 components captured > 95% of variance in vocal tract shapes. These components were interpreted as reflecting (1) Tongue position (40.7%), (2) vocal tract length (34.0%), (3) Body size (9.3%), (4) tongue shape (8.2%), (5) vocal tract curvature (3.4%). Further components explained little variance in shape and did not readily admit to interpretation. These functional principal components (fPCs) can be explored interactively using the companion app to this article, which shows the estimated shape of the vocal tract for any combination of fPC scores (retrieve source code and data from https://osf.io/g59w8/ to run the app locally in R, or view online via https://michelbelyk.shinyapps.io/Variation_in_Vocal_Tract_Morphology/)﻿.

**Figure 2:**
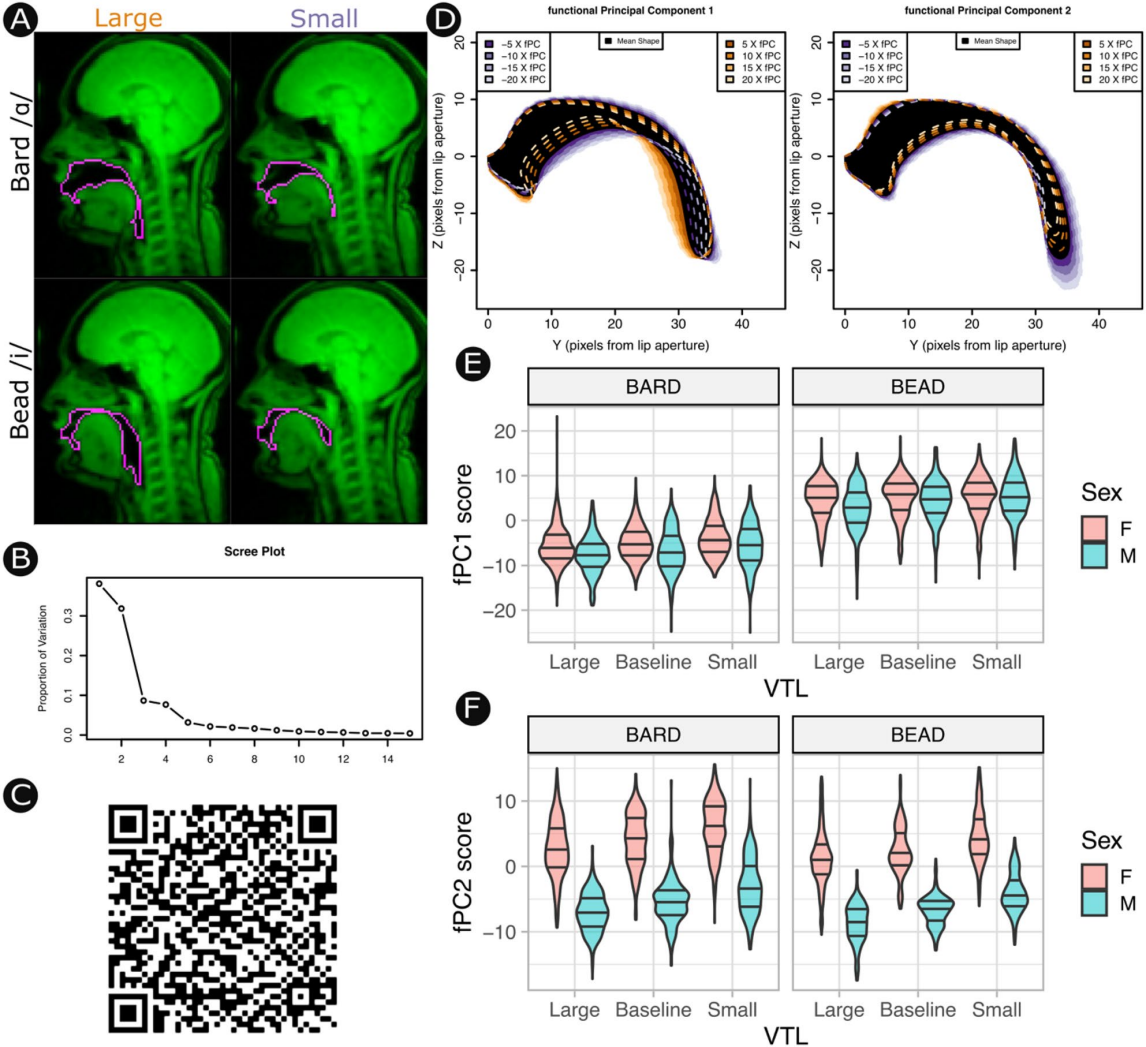
Variation in vocal tract shape. (**A**) Sample traces (pink) of the vocal tract from one speaker producing the vowels sounds in Bard (top) and Bead (bottom) while sounding large (left) or small (right). (**B**) Scree plot demonstrating that ~ 75% of variation in vocal tract shape is captured by functional principal components (fPC) 1 and 2, with fPC 3–5 being of potential interest but relatively small impact. (**C**) An interactive RShiny data visualisation can be accessed by scanning the QR code, or via the following link https://michelbelyk.shinyapps.io/Variation_in_Vocal_Tract_Morphology/. This companion app allows the user to interactively explore combinations of the first five fPCs and view the corresponding vocal tract shapes. (**D**) Static visualisations of fPC1 (left) and fPC2 (right) that provide interpretations for these components. The black area depicts the mean shape of the vocal tract; orange shading and lines indicate the vocal tract shapes that correspond to increasing fPC scores, while purple shading and lines indicate the vocal tract shapes that correspond to decreasing fPC scores. fPC1 has a clear interpretation as a continuum of tongue positions from a back position as is used to produce the vowel /ɑ/ in Bard, to a front position as is used to produce the vowel /i/ in Bead. fPC2 has a clear interpretation as a continuum from a lowered larynx which elongates the vocal tract to a raised larynx with shortens the vocal tract. (**E**) Violin plots of fPC1 scores from all rtMRI frames by word, vocal tract length (VTL) condition, and sex. Horizontal lines mark the median and the interquartile range for each distribution. As expected for a component that loads onto tongue position, fPC1 clearly distinguishes between vowel sounds. (**F**) fPC2 scores. As expected for a component that loads onto vocal tract length, fPC2 clearly distinguishes between the sexes and vocal tract lengthening conditions.

As fPC2 loaded nearly exclusively onto vocal tract length (see Fig. 2) it serves as a measure of laryngeal raising (i.e., vocal tract shortening) and laryngeal lowering (i.e., vocal tract lengthening). A linear mixed model was computed to predict fPC2 scores from the vocal tract condition, the sex of the speaker, and whether they had prior vocal training, with a random slope of vocal tract condition (small vs. large) within speaker. Inference is based on iterative comparisons between full and reduced models following Type III sums of squares. Standardized model coefficients are presented as effect sizes estimates (E)^37^ with 95% confidence intervals (CI). Model assumptions were checked by visual inspection of residuals.

Vocal tract size as quantified by fPC2 was modulated significantly as a function of vocal tract condition (F(2, 49.7) = 68.9, *p* < 0.001; small: E = 2.22, CI = [1.83, 2.61]; large: E = − 1.6, CI = [− 1.86, − 1.0]) and scores were lower overall (i.e., vocal tracts were larger) in male speakers (F(1, 59.9) = 123.4, *p* < 0.001; E = − 9.38, CI = [− 11.15, − 7.62]). There was no difference in fPC2 scores between singers and non-singers at baseline (F(1, 49.9) = 1.61, *p* = 0.21, E = − 1.18, CI = [− 2.85, 0.50]). These findings are consistent with fPC2 as marker of vocal tract length with positive values indexing a smaller vocal tract. Singers modulated the size of their vocal tract more than non-singers (F(2, 49.7) = 4.2, *p* = 0.02; small: E = 1.06, CI = [0.28, 1.9]; large: E = − 0.96, CI = [− 1.77, − 0.14]). Males modulated vocal tract size to a similar degree as females (F(2, 49.7) = 0.67, *p* = 0.51; small: E = 0.29, CI = [− 0.53, 1.10]; large: E = − 0.44, CI = [− 1.30, 0.42]). There was no significant interaction between sex and singing experience (F(1, 49.7) = 0.54, p = 0.59 ; small: E = − 0.87, CI = [− 1.65, 1.76]; large: E = 0.06, CI = [− 2.49, 0.75]).

**Figure 3:**
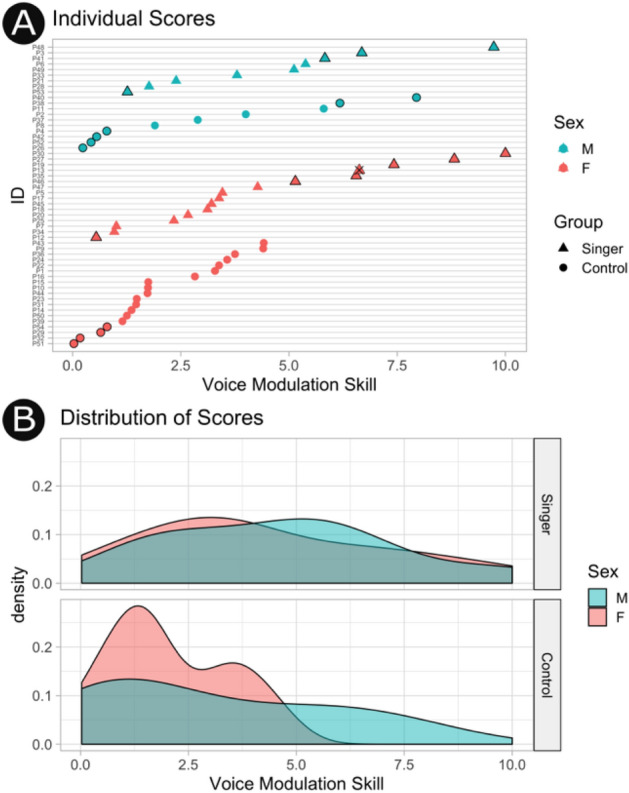
Vocal tract modulation skill. (**A**) Individual vocal tract modulation skill scores (calculated as the difference in median fPC2 scores while imitating larger vs. smaller sounding vocal targets) are plotted for each participant grouped by vocal training and sex and ranked within those groupings. The five highest and lowest ranked speakers are marked by black outlines and compose the good and poor vocal tract modulation skill groups. One participant marked with an x did not consent to their recordings being played to third parties for subsequent experiments and was therefore excluded. (**B**) The distributions of vocal tract modulation skill scores demonstrating an overall advantage for singers despite considerable variation within both groups.

We derived a vocal tract modulation skill score for each speaker in order to quantify vocal tract modulation skill as the difference between median fPC2 skill scores when sounding larger or sounding smaller (see Fig. 3). The score was calculated for each speaker as the difference in median fPC2 scores for imitating larger vs. smaller vocal tracts (median(fPC2_small_) − median(fPC2_large_)). The five highest and lowest ranked speakers were identified as good and poor vocal tract modulation skill groups in subsequent analyses. Vocal tract modulation skill scores were significantly greater in singers than non-singers (F(1, 50) = 9.87, *p* = 0.003, E = 2.23, CI = [0.66, 3.9]). There was no clear difference between the sexes, (F(1,50) = 0.26, *p* = 0.61, E = 1.1, CI = [− 3.42, 2.03]) nor a sex-by-group interaction (F(1,50) = 1.12, *p* = 0.29, E = − 0.51, CI = [− 0.86, 2.87]).

### Experiment 1: speech acoustics

#### Prioritisation of acoustic measures

**Figure 4:**
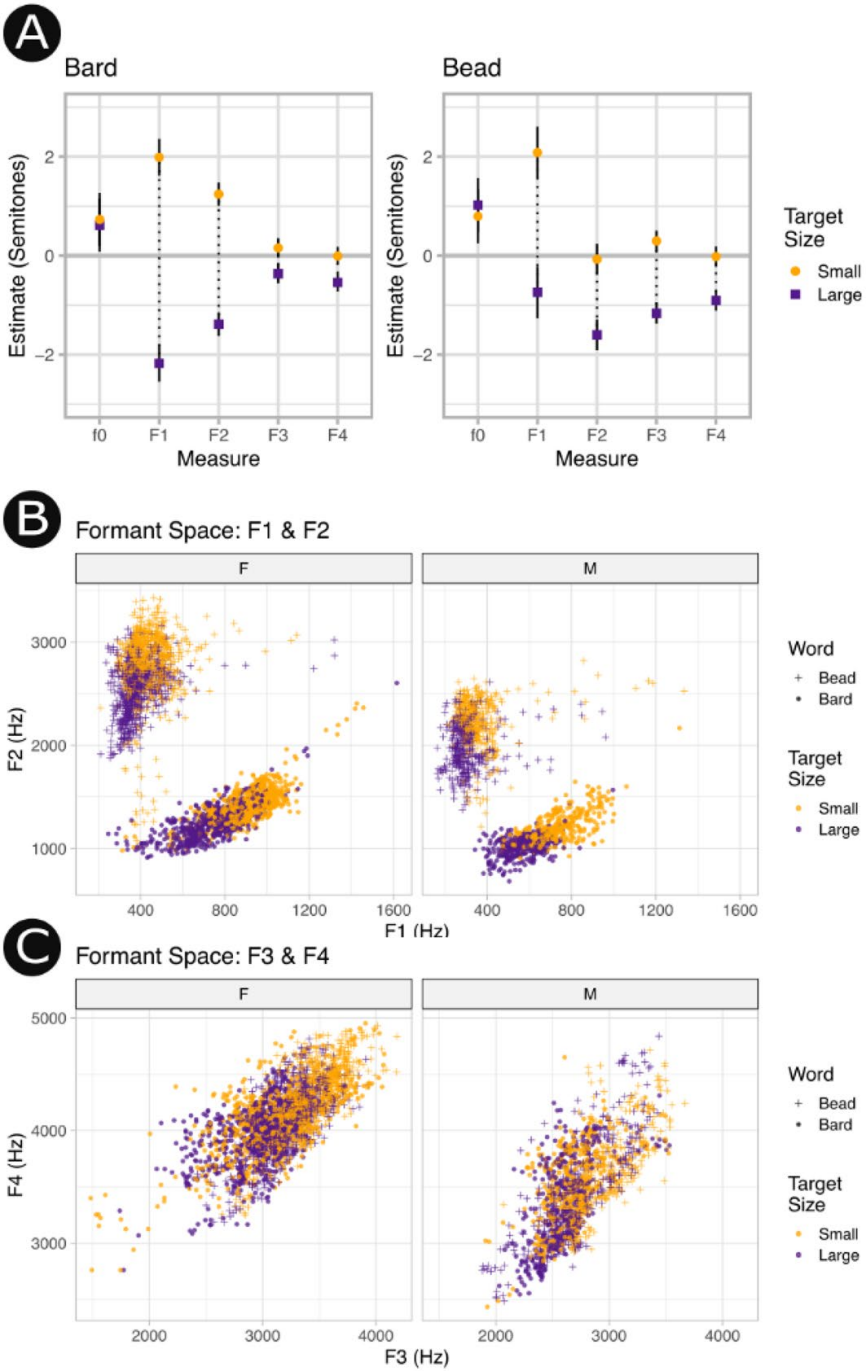
Exploration of acoustic measures. (**A**) Contrasts of acoustical parameters presented for the words Bard (vowel /ɑ/) and Bead (vowel /i/) while sounding large or small. Measurement pairs are joined by dotted lines to facilitate comparison. Solid lines indicate 95% confidence intervals. The two most speech-relevant acoustical measures (F1, F2) were modulated by speakers with F1 being modulated most strongly and reliably. F3 was modulated weakly and only for the carrier word Bead the vowel in which is known for the unusual property of being partly encoded by F3. (**B**) The formant space of F1 and F2 demonstrate the canonical separation of these two vowels. Strong upward frequency shifts are evident when speakers attempted to sound small (orange) and downward frequency shifts when speakers attempted to sound large (purple). (**C**) The formant space of F3 and F4 showing poor separation between attempts to sound small or large. See Supplementary Materials 1 for an interactive data visualisation of F1, F2, and f0 space demonstrating the relative independence of vocal tract modulation skill from vocal pitch modulation.

**Figure 5:**
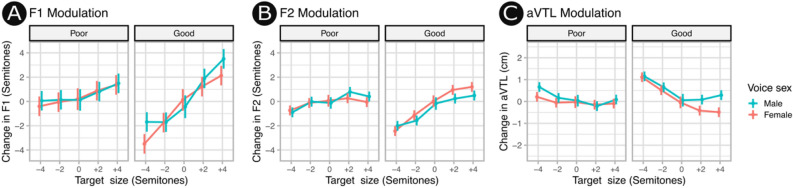
Modulation of (**A**) F1, (**B**) F2, (**C**) aVTL for good and poor vocal modulators as identified from rtMRI. Good vocal modulators of both sexes made greater changes to the acoustics of their voice than poor vocal modulators, particularly for F1. See additional analyses reported in Supplementary Materials 2.

An initial series of linear mixed models with fixed effect predictors of vocal tract length modulation and random intercepts of speaker identity were fit separately for the first four speech formants (F1, F2, F3, F4), apparent Vocal Tract Length, (aVTL), and the fundamental frequency of the voice (f0). For comparability with the rtMRI data only the most extreme conditions (± 4 semitones) and the baseline were included in the model. These models demonstrated that when speakers attempted to sound larger or smaller, they primarily modulated the acoustical measures that are most relevant to human speech sound production (see Fig. 4). In particular, F1 was strongly modulated across carrier words and F2 was modestly modulated depending on carrier word, while F3 and F4 were only minimally modulated. Intermediate conditions (± 2 semitones) are additionally included in Fig. 5 for illustration.

#### Correlation with vocal tract modulation skill score

**Figure 6:**
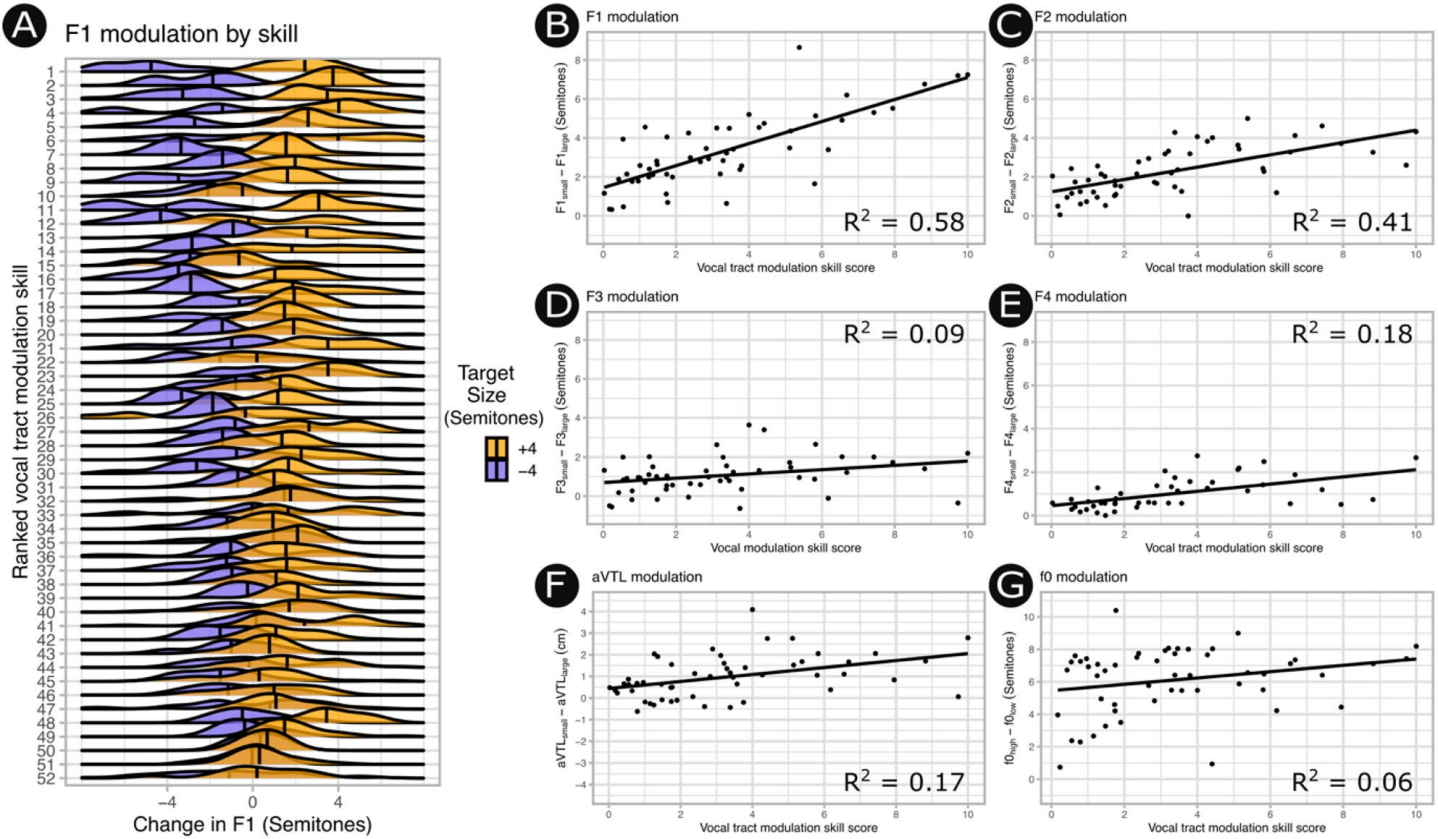
Modulation of speech acoustics according to vocal tract modulation skill. (**A**) Ridgeline plot showing change in F1 (x-axis) sounding small (orange) or large (purple) for each participant (y-axis). Participants are ranked in descending order according to their vocal tract modulation skill score as derived from rtMRI measurements. Larger separations between distributions for small versus large indicate greater modulation of vocal acoustics. (**B**) A strong Pearson correlation between vocal tract modulation skill as derived from rtMRI (x-axis) and acoustical modulation (y-axis) in F1 demonstrates that speakers with a greater ability to change the size of their vocal tract produced larger changes in the quality of their voice. (**C**–**E**) Weaker correlations are evident for the higher formants, and (**F**) correspondingly the composite measure aVTL correlates only modestly. (**G**) Vocal tract modulation skill was relatively independent from pitch matching ability.

Acoustic modulation scores were calculated for each acoustic measurement as the change in the median value between the ± 4 semitone conditions (see Fig. 5). Acoustic modulation (see Eq. 3) was correlated with vocal tract modulation skill (see Eq. 1) across all formants tested, with the strongest correlations observed for the lower, more speech-relevant, formants (F1: R^2^ = 0.58, t(50) = 8.39 *p* < 0.001; F2: R^2^ = 0.41, t(50) = 5.92, *p* < 0.001; see Fig. 6). This is consistent with the broader finding that speakers modulated F1 and F2 more strongly in general (see Fig. 4). The higher formants were more modestly correlated with vocal tract modulation skill (F3: R^2^ = 0.09, t(50) = 2.25, *p* = 0.015; F4: R^2^ = 0.18, t(50) = 3.34, *p* < 0.001), as was aVTL, which is a composite derived from F1-F4 (aVTL: R^2^ = 0.17, t(50) = 3.25, *p* = 0.001). Notably, the ability to accurately imitate vocal pitch was only weakly correlated to vocal tract modulation skill, suggesting that vocal tract modulation may be partially independent of singing ability (f0: R^2^ = 0.06, t(50) = 1.78, *p* = 0.04).

### Experiment 2: perceptual height judgements

**Figure 7:**
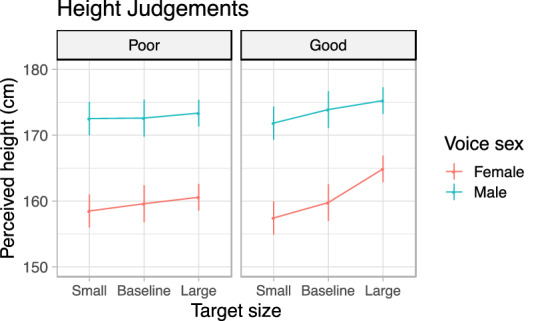
Modulation of perceived height. Poor vocal modulators (left) had little to no influence on their perceived height, whereas good vocal modulators (right) were reliably perceived as being shorter or taller. This was particularly evident among female good vocal modulators.

Linear mixed models evaluated the effects of speaker group (good vs. poor vocal modulators), vocal tract condition (± 4 semitones vs. baseline), and speaker sex (male vs. female). Model estimates are presented in Fig. 7.

#### Basic effects of modulation

Speakers were rated as sounding taller or shorter when they modulated the length of their vocal tracts (F(219.1) = 27.9, *p* < 0.0001; small: E = − 1.40, CI = [− 2.14, − 0.66]; large: E = 2.1, CI = [1.38, 2.73]), and along with changes in the pitch of their voice (F(1, 36.7) = 234.4, *p* < 0.0001; E = − 8.02, CI = [− 9.16, − 6.87]). The influence of voice pitch was considerably reduced when paired with vocal tract modulations in either direction relative to baseline (F(2, 13,493.7) = 64.8, *p* < 0.0001; small: E = 4.96, CI = [3.87, 6.04]; large: E = 5.76, CI = [4.68, 6.84]).

#### Vocal tract modulation skill

Good vocal modulators had a greater influence over their perceived height by modulating the length of their vocal tract (F(2, 19.1) = 9.2, *p* = 0.002; small: E = − 1.60, CI = [− 3.08, − 0.12]; large: 2.38, CI = [1.03, 3.73]) and a surprisingly moderate advantage in modulating vocal pitch (F(1, 36.7) = 4.3, *p* = 0.046; E = − 2.85, CI = [− 5.15, − 0.56]). A small-three way interaction between modulation skill group, vocal tract length condition, and vocal pitch (F(2, 13,493.7) = 4.2, *p* = 0.015; small: E = 2.23, CI = [0.05, 4.40]; large: E = 2.73, CI = [0.56, 4.89]) showed that the perceived heights of good vocal modulators were less strongly determined by vocal pitch. The latter effect may result from poor vocal modulators relying more strongly on vocal pitch to compensate for limitations in their ability to modulate vocal tract length.

#### Sex differences

For male speakers, vocal tract modulation had a reduced impact on perceived height, primarily driven by a reduced ability to sound large (F(2, 19.1) = 5.0, *p* = 0.018; small: E = 0.66, CI = [− 0.83, 2.14]; large: E = − 1.99, CI = [− 3.34, − 0.64]). Likewise, the influence of vocal pitch modulation in males was considerably reduced (F(1, 36.7, = 30.3, *p* < 0.0001, E = 6.4, CI = [4.11, 8.70]). However, a greater interaction between vocal tract lengthening and vocal pitch modulation was also observed in males (F(2, 13,493.7) = 9.79, *p* < 0.0001; small: E = − 4.76, CI = [− 6.94, − 2.59]; large: E = − 4.88, CI = [− 7.05, − 2.72]), suggesting that vocal tract lengthening was not as strongly dominant over vocal pitch in male voices.

#### Vocal tract modulation skill by sex interactions

There was ambiguous evidence for a sex difference in the advantage of good vocal modulators in the use of vocal tract length (F(2, 19.1) = 3.3, *p* = 0.058; small: E = − 0.75, CI = [− 3.71, 2.21]; large: E = − 3.54, CI = [− 6.24, − 0.84]) and no evidence of sex differences in the use of vocal pitch (F(1, 36.7) = 2.2, *p* = 0.15, E = 5.48, CI = [0.89,10.07]). However, the complex interaction between vocal tract condition, vocal pitch, and vocal tract modulation skill group did differ between the sexes (F(2, 13,493.7) = 4.1, *p* = 0.018; small: E = − 6.02, CI = [− 10.37, − 1.67]; large: E = − 5.31, CI = [− 9.64, − 0.98]) such that being a good vocal modulator who is male did less to reduce reliance on vocal pitch cues than being a good vocal modulator who is female.

## Discussion

Speakers varied considerably in their ability to manipulate their vocal size, and this trait was consistent from the dynamic physiology of the vocal tract, through vocal acoustics, to the impressions that were formed by listeners. Speakers who were better able to modulate the length of their vocal tract produced greater changes in the acoustics of their voice, and correspondingly were more able to manipulate how they are perceived in the ear of the listener. Moreover, individual variation in this skill ranged so extensively that the best performing speakers matched target vocal sizes with near perfect fidelity on average, while the worst performing speakers demonstrated little ability to modulate vocal size.

In the absence of modulation, the apparent size of the voice follows an allometric relationship with the size of the speaker’s body^6,22,38^. This allometry is likely to hold for poor vocal modulators, whose vocal tract sizes are relatively stable. However, strong vocal modulators may selectively and volitionally exaggerate their vocal size to deviate from allometric expectations. To the extent that vocal size contributes to socially relevant cues such as vocal attractiveness, social dominance, and authoritativeness, good vocal modulators may also have an advantage in influencing listeners impressions of them as befitting social context.

### Constraints on vocal tract modulation

There is a tension between the interests of the speaker in exaggerating socially desirable attributes of their voice, and the interest of the listener in achieving an honest assessment of the speaker. Evidence from a range of species suggests that signalling systems are usually only maintained when communication is mostly honest^39,40^. This tendency towards honesty comes from systematic constraints on the effectiveness of deception rather than abstinence from dishonest signalling^41,42^. For example, humans—like other animals—prefer partners whose bodies and faces are symmetrical because symmetry is a cue to the individual’s ability to cope with environmental and genetic stressors^43,44^. Vocal attractiveness correlates with bilateral symmetry, suggesting that the voice may also act as an honest signal of quality^45,46^. However, it remains to be understood how a signal as dynamic as the human voice is constrained to remain honest such that it continues to be informative to listeners.

We observed sex differences in voice modulation ability that may reflect constraints at the level of the vocal tract. Male speakers were less effective at lengthening their vocal tracts, in emphasizing lower formant frequencies, and thereby in being perceived as larger (i.e., masculinising their voices). Females were less effective at shortening their vocal tracts, in emphasizing higher formant frequencies, and thereby in being perceived as smaller (i.e., feminising their voices). These limitations may stem from constraints on the anatomy of the vocal tract. During the pubertal stage of human development, the larynx descends and the vocal tract becomes longer. The descent of the male larynx is more extreme, resulting in a disproportionately longer vocal tract and larger sounding voice at baseline^23,28^. This is consistent with sexual dimorphism observed across a wide range of mammals^47^, which tends to enhance the vocal size of males. The lower resting position of the male larynx may limit the available downward range of motion, while the higher resting position of the female larynx may limit the available upward range of motion. We suggest that the sexual dimorphism in the resting position of the larynx places sexually dimorphic constraints on vocal tract modulation. These constraints on movement limit the extent to which speakers can exaggerate vocal cues which contribute to vocal attractiveness and may help to maintain the correlation between voice cues and sexually dimorphic features of the body that are associated with mate quality^5,14^.

Speakers primarily modulated F1 and F2, and had relatively little impact on higher formants. This is consistent with previous observations that higher formants are a more robust source of information about vocal tract length^48^. While our findings demonstrate that listeners are indeed influenced by the formants which are most strongly under the speaker’s control, there is recent evidence to suggest that listeners may monitor for deceptive voice cues and adapt their perceptions of the speaker accordingly^7^. Together, these findings lead to the prediction that listeners may place less weight on the formants over which speakers exercise dynamic control and greater weight on those which are liable to be stable.

### Dynamic control over vocal tract length

It has previously been hypothesised that voice modulation may have contributed to selective pressure for adaptations in the brain that support enhanced control over the voice^38,49^. If individuals with nervous systems that promote greater vocal flexibility are more able to manipulate their social environment, then the prevalence of such traits should increase in the population. The human larynx motor cortices (LMCs), which control the muscles of the larynx, differ markedly in humans from the homologous system in other primates^50^. Among other functions, the LMCs are involved in raising or lowering the larynx^51^ and participate in broader brain networks^52^ involved in characteristically human modes of communication such as speaking and singing^53,54^, as well as expressing emotions^55,56^. Vocal trait modulation similarly engages this system, alongside a broader network of brain areas associated with social reasoning^57^.

Adjacent to the LMCs is a putative larynx somatosensory cortex, which is enhanced in highly trained Opera singers^58,59^ and may mediate the improved vocal modulation skill of singers^36^. In the present study, there was a tendency for greater vocal tract modulation skill among highly trained singers. However, it cannot be determined from the present data whether this advantage was due to their training as vocalists^33–35^, or whether individuals with strong control over the vocal apparatus are more likely to self-select into vocal training.

### Limitations

Formant measurements were collected using the Linear Predictive Coding algorithm of Burg (LPC-Burg) as implemented in praat^60^. This method produces reliable estimates of formant frequencies under most conditions, but may become imprecise when particularly low formants are estimated from particularly high-pitched voices. In particular, this approach is insensitive F1 values below the value of f0, effectively inducing a measurement floor^61^. This may have affected one set of conditions in the present study in which females vocalised with raised f0 while speaking the carrier word “bead” (i.e., a vowel acoustically encoded by low F1) and sounding large (i.e., further lowering F1). An examination of the individual measurements suggests that a small number of observations in this condition are likely to have been impacted by the f0/F1 measurement floor (see Supplementary Materials 3). However, no bias was evident at the level of within-speaker means and performance was broadly similar while females spoke with a high pitch (which is vulnerable to floor effects) or with a low pitch (which is not vulnerable to floor effects). Moreover, we note that female speakers were observed to be more effective at lowering their formants than raising them. If LPC-Burg related measurement error has impacted the present findings, its effect will have been to produce a small underestimation of the sex differences reported by the present study.

## Conclusions

There is considerable inter-individual variation in the ability to modulate socially relevant aspects of the voice. This variation spanned the causal pathway from the dynamic physiology of the vocal tract, through vocal acoustics, to the impressions formed by listeners and ranged from nearly incapable to nearly perfect performance within the range of vocal modulations that were tested. Vocal modulation ability may nonetheless be sexually dimorphic, such that speakers have the greatest difficulty exaggerating sex-typical vocal features. Further research is needed to understand whether this variation is an inherited trait of the individual or the result of life experiences such as vocal training.

## Materials and methods

### Experiment 1: speech production

#### Participants

A total of 57 adults (20 male; Mean age = 24.7 years, s.d. = 5.7, range = 19–43 years) with healthy hearing and no neurological illness (self-reported) completed the study. Twenty-seven participants (10 male; Mean age = 27.5 years, s.d. = 6.4, range = 20–43 years) were highly trained singers, with the primary selection criterion that they should have studied voice as 1st study at university/music performance college. The remaining 30 participants (10 male; Mean age = 22.1 years, s.d. = 3.4, range = 19–35 years) formed a control group. All participants completed a questionnaire on music and language experience—this showed that the singers had on average 16.3 years of training in voice (range = 5–35 years). MRI data from three participants were discarded due to technical issues during scanning.

#### Stimuli

Participants were recorded saying the words “bead” and “bard” 5 times each with the instruction to produce these at a normal pitch and with a slightly longer than natural duration—this was in order to obtain a sufficiently steady state portion of the vowel for imitation and acoustic/vocal tract analysis in the main experiment. These words were chosen because they are (1) framed by the same stop consonants, which facilitate the detection of sound onset and offset, and (2) are distinguished by the front vowel /i/ or the back vowel /ɑ/ which are easily distinguished by tongue placement (see Fig. 2E,D) and cover two extremes of the human vowel space.

The experimenter selected one representative token of each word, aiming for a duration of 0.6–0.8 s and a good voice quality (without, for example, vocal fry, which introduces distortions in the synthesis of target stimuli). The two selected tokens were then transformed into f0- and VTL-modulated targets using a modified version of a procedure developed by Chris Darwin at the University of Sussex (http://www.lifesci.sussex.ac.uk/home/Chris_Darwin/Praatscripts/VTchange) that allows adjustment of the f0 and formant frequencies as ratios of the original stimulus values. A central, “baseline voice” version of each word was produced, in which the formants were unchanged but the f0 was shifted 2 semitones upward from the original (to allow for the generation of lower-pitch targets that would not go beyond the speaker’s natural range). In addition, there were 16 modified versions of “bead” and “bard”, in which the VTL and f0 were further adjusted relative to the “baseline voice”, either by shifting both the f0 and VTL by 2 or 4 semitones in the same direction (e.g. + 2 f0, + 2 formants; − 4 f0, − 4 VTL), or in opposite directions (e.g., + 2 f0, − 2 VTL; − 4 f0, + 4 formants). This produced 2 “axes” along which voice targets varied, in 2-semitone steps along each axis (see Fig. 1). Together these conditions spanned a continuum of biologically likely voice conditions from low-pitched and large sounding to high-pitched and small sounding, as well as a biologically unlikely continuum from low-pitched and small sounding to high-pitched and large sounding.

#### Imitation task

Participants listened to recordings of their voice that had been manipulated to have higher or lower vocal pitch and with formants shifted such that they sounded smaller or larger. The participant viewed a short presentation (lasting approx. 4 min) in Microsoft PowerPoint (Microsoft Corporation, Albuquerque, NM), in which they were introduced to examples of modified stimuli of the type used in the experiment (presented over headphones) and instructed how to perform the imitation task. The presentation can be found in the supporting data for this paper (https://osf.io/6pqkt/).

Participants completed an audio recording session in which they produced imitations of all 18 voice targets ((1 baseline voice + 8 modulated targets) × 2 words). Stimulus presentation and data collection was performed using MATLAB with the Psychtoolbox extension^62^. Each condition was presented in two non-consecutive blocks of 5 trials each in pseudorandomized order (2 blocks × 5 trials × 18 conditions = 180 trials). Participants were given the opportunity for a short break every 6 blocks.

Participants repeated the imitation procedure in a separate session at an MRI scanner. Participants imitated the baseline voice condition and the 4 most extreme voice transformations (i.e., the ± 4 semitone endpoints of the axes tested in the audio recording session). All stimuli were delivered through MR-compatible earbuds (Sensimetrics S14; Sensimetrics Corporation, Gloucester, MA) and task performance was recorded and verified with a fibre-optic microphone (FOMRI-III; OptoAcoustics Ltd, Or Yehuda, Israel). All stimuli were presented via the Psychophysics toolbox running in MATLAB, with back projection for presentation of visual stimuli.

Participants completed 3 rtMRI runs (126 s each), interspersed with runs of functional MRI (data reported elsewhere^36^). Each ((1 baseline voice + 4 modulated targets) × 2 words) was performed in 10 blocks of 4 consecutive trials to a total of 40 repetitions each. Blocks were presented in pseudorandom order. Each trial began with delivery of an audio stimulus and a visual prompt (“Listen”), followed after 1.2 s by a prompt to imitate (“Repeat”) and a 1.5 s gap in which the participant produced their imitation.

Real-time MRI data were fast gradient echo images collected on a Siemens 3 T TIM Trio scanner; flip angle: 5°; TE/TR: 1.25/3.2 ms; GRAPPA factor 2; partial-Fourier: 75%; FOV 220 × 274 mm^2^; 2.5 × 2.5 × 10.0 mm^3^ spatial and 125 ms temporal resolution (8 frames per second [f.p.s.]). Pilot experiments showed that we could obtain adequate numbers of frames during steady-state phonation when sampling at 8 f.p.s., to enable us to index articulator positioning for the vowels.

#### Analysis: real-time MRI

We developed a novel tool for semi-automatically extracting the shape of the vocal tract from rtMRI data using spatially constrained tissue classification. Each rtMRI frame was registered to a single representative image from one participant to ensure that images shared a common position and orientation. The transformation matrix describing a rigid body transform was estimated from images that included only containing static structures such as the skull and vertebrae, and excluding the labile structures of the vocal tract such as the lips, tongue, velum, pharynx, and larynx. The rotation and translation parameters estimated from the static structures was then applied to the full image, including labile structures. This procedure ensured that vocal tract images shared a common position and orientation to facilitate comparison.

The approximate location of the vocal tract within the rtMRI series was estimated by identifying high variance pixels, since alternation between high intensity (soft tissue) and low intensity (air) is a characteristic of vocal tract pixels. An informed analyst (MB) then manually adjusted this estimate to create a mask that identified pixels that may sometimes contain vocal tract. These pixels were then subject to simple tissue classification based on the high degree of contrast between air and soft tissue in T1-weighted images. These tissue masks were then converted to outlines, manually inspected, and corrected for tissue classification errors where necessary.

Vocal tract traces were analysed using functional principal components analysis (fPCA)^63,64^ in R (v3.6.1)^65,66^ following a method we have previously demonstrated on outlines of the tongue during whistling^67^. Functional PCA explores patterns of variation in the shapes of functions around a mean shape. Much like discrete PCA, fPCA seeks principal components that maximize variation between observations^68,69^. The principal components of discrete PCA are eigenvectors that map each component back onto a set of discrete variables. ﻿Similarly, the principal components of functional PCA are eigenfunctions that map each component back onto variations in shape. Applied to the two-dimensional coordinates of the outline of the vocal tract, this approach provides an empirical means of studying changes in vocal tract shape.

One component identified in this analysis loaded strongly onto vocal tract lengthening or shortening, with little to no change evident elsewhere in the vocal tract. This component can be used to quantify larynx raising and lowering. For each participant, we calculated a vocal tract modulation skill score (see Eq. 1) as the median component score when sounding large minus the median component score while sounding small.

1 $${\text{VT}}\;{\text{skill}}\;{\text{score}} = {\text{median}}\left( {{\text{fPC}}2_{{{\text{small}}}} } \right) - {\text{median}}\left( {{\text{fPC}}2_{{{\text{large}}}} } \right)$$

The 5 speakers with the highest vocal tract modulation skill scores for each sex were identified as good vocal modulators while the 5 speakers from each sex with the lowest scores were identified as poor vocal modulators for subsequent analyses. Audio recordings from the good and poor vocal modulators were selected as stimuli for subsequent perceptual experiments.

#### Analysis: voice acoustics

Acoustical measurements f0 (the acoustical correlate of voice pitch) and speech formants F1-F4 were extracted from audio recordings produced in a sound attenuated booth and used to derive a measure of acoustical vocal tract length (aVTL)^70^. Acoustical measurements were not taken from the MRI session to avoid contamination from imaging-related acoustical artefacts. Audio recorded from the scanning session was used only to identify time points during which vocalisation occurred.

Acoustic measurements were taken from the vocalic portion of each recorded word. Praat software^60^ was used to measure f0 and the centre frequencies of formants F1–F4. Formants were estimated using linear predictive coding (LPC) Burg estimation. Formants values were used to derive a composite measure called apparent vocal tract length (aVTL)^70^ which pools information across formant bands. Analyses of formants were based on formant modulation in semitones relative to baseline (see Eq. 2).

2 $${\text{Fx}}\_{\text{modulation}} = \log 2\left( {{\text{Fx}}_{{{\text{observed}}}} /{\text{Fx}}_{{{\text{baseline}}}} } \right)*12$$

Formant modulation was calculated separately for each participant and each carrier word. A simplified linear mixed model was fit to assess the degree to which each formant was modulated, with a fixed effect predictor of vocal tract condition (± 4 semitones and baseline) and a random intercept of speaker.

An acoustic modulation score was calculated following a similar procedure as the vocal tract modulation skill score derived from rtMRI (see Eq. 3). Pearson correlations were calculated between vocal tract modulation scores and acoustic modulation scores.

3 $${\text{Acoustic}}\;{\text{skill}}\;{\text{score}} = {\text{median}}\left( {{\text{Fx}}\_{\text{modulation}}_{{{\text{small}}}} } \right) - {\text{median}}\left( {{\text{Fx}}\_{\text{modulation}}_{{{\text{large}}}} } \right)$$

### Experiment 2: perceptual height judgements

#### Participants

One-hundred sixty-one participants (80 female, ages 18–40) were recruited from Prolific, an online participant recruitment platform. Recruitment instructions required that participants complete the experiment in a quiet space on a desktop or laptop computer while wearing headphones. Recruitment was restricted to participants who self-identified as having normal or corrected-to-normal vision, no hearing difficulties, speak English as a first language, have no ongoing mental health conditions, and had an excellent approval rating (> 90) from the recruitment platform.

#### Stimuli

Stimulus recordings were selected from the 5 speakers with the highest vocal tract modulation skill scores for each sex (good vocal modulators), and the 5 speakers with the lowest vocal tract modulation skill scores (poor vocal modulators). Recordings were selected from the unmodulated baseline voice condition as well as the four conditions with most extreme voice modulations (± 400 cents of f0 and ± 400 cents of formants). Within each stimulus category, a representative token was selected by finding the recording with the smallest deviation from the within-condition median f0, F1, and F2. This procedure selected 100 stimulus recordings from male speakers and 100 from females.

#### Task

Listeners were randomly assigned to rate either male or female voices. Recordings were presented one at a time and listeners were instructed to guess the heights of speakers using a slider that ranged from the 1st to the 99th percentile of height for females (145 to 180 cm) and males (155 to 190 cm), respectively (https://dqydj.com/height-percentile-calculator-for-men-and-women/).

#### Analysis: perceptual height judgements

Height judgments were analysed by linear mixed models with a form similar to those for the acoustic data, save that individual differences between raters were also modelled by random intercepts, and the larger number of observations on each speaker made it possible to include random slopes within speaker as preferred by Akaike Information Criterion. The model took the form:

$$\begin{aligned} & Perceived\_height\sim VTL\_skill\_group*VTL\_condition*f0\_modulation* \, Voice\_sex + \left( {1/Listener} \right) \\ & \quad + \;\left( {1 + f0\_modulation + vtl\_condition/Speaker} \right) \\ \end{aligned}$$

### Research ethics

Experiment 1 was approved by the Psychology Department Ethics Committee at Royal Holloway, University of London. Experiment 2 was approved by the Department of Speech, Hearing and Phonetic Science Ethics Committee at University College London [SHaPS-2019-CM-030]. All participants provided informed consent and all research was carried with the approved guidelines.

## Supplementary Information

Supplementary Information 1.

Supplementary Information 2.

Supplementary Information 3.

## Data Availability

Processed data and analysis code are archived at https://osf.io/g59w8/?view_only=0102e14963984d9b8ea2606dbdd41558.
