# Supplementary material for: Individual differences in vocal size exaggeration
Source: Sci Rep. 2022 Feb 16;12:2611. doi: 10.1038/s41598-022-05170-6 (PMC8850436; doi:10.1038/s41598-022-05170-6)
Supplement: Supplementary file 2 — Supplementary Information 2. [file 41598_2022_5170_MOESM2_ESM.pdf]

Supplementary analysis of acoustic measures

All analyses are based on linear mixed models computed in R (Bates et al., 2015; R Core Team, 2019). Inference is based on iteratively comparing full and reduced models following Type III sums of squares. All formants are presented as modulations in semitones relative to within subject medians at baseline [e.g., Fx_modulation = log2($Fx_{observed}$/median($Fx_{baseline}$)*12]

Model selection informed by the Akaike Information Criteria (AIC) took the form:

*Fx_modulation~VTL_skill_group*VTL_condition*f0_modulation*Voice_sex+(1|speaker)*

VTL_skill_group categorizes speakers into Good or Poor vocal modulators with Moderate vocal modulators taken as baseline. For comparability with data from the rtMRI session only measurements from the most extreme conditions (+/- 4 semitones) relative to the unmodulated condition (0 semitones). f0_modulation is a continuous covariate the vocal pitch of each utterance, regardless of the pitch of the target stimulus.

Standardized estimates are presented as effect sizes following Gelman (2008). Fixed effect predictor variables were scaled by twice their standard deviation such that parameter estimates within the model serve as standardized measures of effect magnitudes.

A narrative description is provided for F1. Summary tables are provided for higher formants.

Analysis of first formant (F1) modulations

Good vocal modulators made larger changes to the acoustics of their voice than poor vocal modulators. Males were more effective than females at raising formants in simulation of a smaller body size, while females were more effective than males at lowering formants to simulate a larger body size. This sex difference was most evident among the good vocal modulators, presumably reflecting differences in range of movement available to either sex. Statistics are reported along with standardized estimates (SE) and 95% confidence intervals (CI). Positive values indicate increased apparent heights, while negative values indicate decreased apparent height.

Basic effects of modulation

Speakers modulated F1 according to target vocal size ($F(2, 1827.2) = 386.6$, $p < 0.0001$; small: E =2.13, CI = [1.78, 2.49]; large: E =-1.40, CI = [-1.04, -1.76]), independently of target vocal pitch ($F(1, 1826.2) = 2.0$, $p = 0.15$, E =-0.83, CI =[-2.80, 1.13) and with no interaction of size and pitch ($F(2,1928.1) = 0.2$, $p = 0.82$; small: E =0.46, CI = [-1.53, 2.45]; large: E =0.55, CI = [-1.44, 2.55]).

Differences between vocal modulation skill groups

Good vocal tract modulators made greater changes to F1 than poor vocal tract modulators ($F(2, 1927.2) = 114.3$, $p < 0.001$; small: E =1.74, CI = [1.03, 2.45]; large: E =-2.11, CI = [-1.40, -2.82]), and particularly when the target vocal pitch was incongruent with the target vocal tract length (e.g., small sounding with a low pitch) ($F(1, 1928.2) = 12.2$, $p = 0.0004$, E =-3.42,

CI = [-7.33, 0.49]). A significant interaction between vocal modulation skill group, target vocal tract length, and target vocal pitch modulation suggests that this pairing of vocal pitch and vocal tract modulation varied by condition (F(2, 1928.1) = 6.5, p = 0.001; small: E =2.3, CI = [-1.60, 6.35]; large: E =0.66, CI = [-3.28, 4.64]), though a separate examination of the magnitudes of these effects for sounding smaller or larger was not compelling.

Sex differences

Male speakers modulated F1 more when sounding small but less when sounding large (see Figure 5) creating a bias towards higher formant values (F(2, 1927.2) = 8.6, p = 0.0002; small: E =1.16, CI = [0.45, 1.88]; large: E =1.52, CI = [0.80, 2.23]). Males modulated F1 more when vocal size cues conflicted with vocal pitch cues (F(1, 19.28.3) = 12.7, p = 0.004, E =-4.22, CI = [-0.28, -8.17], although this tendency may have varied across conditions (F(2, 1928.1) = 3.4, p = 0.03; small: E =3.16, CI = [-0..84, 7.17]; large: E =2.07, CI = [-1.92, 6.07]).

Group by sex interactions

The performance advantage of good vocal modulators was also affected by the male speakers' bias towards small-sounding vocal modulations (see Figure 5); male good vocal modulators performed better than female good vocal modulators when they raised the larynx to sound smaller, but less well when they lowered the larynx to sound larger (F(2, 1927.2) = 4.3, p 0.01; small: E =1.97, CI = [0.55, 3.40]; large: E =2.05 , CI = [0.63, 3.48]). The tendency for good vocal modulators to shift F1 more when vocal size cues conflicted with vocal pitch cues was evident among males F(1, 1928.2) = 9.84, p = 0.002, E =-10.00, CI = [-2.15, -17.87], and did not appear to interact with the direction of size modulation (F(2, 1928.1) = 2.59, p = 0.07; small: E =9.0, CI =[1.02, 16.99]; large: SE =8.14 , CI = [0.19, 16.11]).

Analysis of second formant (F2) modulations

Predictors	Estimate	Std. Error	df	t value	Pr(>|t|)
(Intercept)	-0.06	0.14	54.69	-0.40	0.69
c.Skill_class	-0.06	0.29	54.48	-0.19	0.85
VTL_condition-4	-1.47	0.12	1927.48	-12.35	0.00
VTL_condition+4	0.59	0.12	1927.59	4.97	0.00
z.f0_modulation	-0.24	0.66	1928.16	-0.36	0.72
c.Sex	-0.21	0.29	54.84	-0.74	0.47
c.Skill_class:VTL_condition-4	-1.36	0.24	1927.48	-5.73	0.00
c.Skill_class:VTL_condition+4	0.70	0.24	1927.59	2.95	0.00
c.Skill_class:z.f0_modulation	-0.51	1.31	1928.17	-0.39	0.70
VTL_condition-4:z.f0_modulation	0.25	0.67	1928.22	0.37	0.71
VTL_condition+4:z.f0_modulation	0.96	0.67	1928.08	1.43	0.15
c.Skill_class:c.Sex	-0.05	0.57	54.62	-0.09	0.93
VTL_condition-4:c.Sex	0.35	0.24	1927.48	1.47	0.14
VTL_condition+4:c.Sex	0.07	0.24	1927.59	0.30	0.77
z.f0_modulation:c.Sex	-0.88	1.32	1928.16	-0.66	0.51
c.Skill_class:VTL_condition-4:z.f0_modulation	0.18	1.33	1928.24	0.13	0.89
c.Skill_class:VTL_condition+4:z.f0_modulation	-0.30	1.33	1928.10	-0.22	0.82
c.Skill_class:VTL_condition-4:c.Sex	0.61	0.48	1927.48	1.27	0.20
c.Skill_class:VTL_condition+4:c.Sex	-1.12	0.48	1927.59	-2.35	0.02
c.Skill_class:z.f0_modulation:c.Sex	-2.10	2.63	1928.17	-0.80	0.43
VTL_condition-4:z.f0_modulation:c.Sex	0.91	1.34	1928.21	0.68	0.49
VTL_condition+4:z.f0_modulation:c.Sex	0.76	1.34	1928.08	0.57	0.57
c.Skill_class:VTL_condition-4:z.f0_modulation:c.Sex	1.11	2.66	1928.24	0.42	0.68
c.Skill_class:VTL_condition+4:z.f0_modulation:c.Sex	1.78	2.67	1928.11	0.66	0.51

Analysis of third formant (F3) modulations

Predictors	Estimate	Std. Error	df	t value	Pr(>|t|)
(Intercept)	0.04	0.10	69.13	0.45	0.66
c.Skill_class	-0.01	0.20	68.83	-0.06	0.95
VTL_condition-4	-0.78	0.09	1927.56	-8.92	0.00
VTL_condition+4	0.19	0.09	1927.71	2.18	0.03
z.f0_modulation	0.25	0.48	1928.42	0.51	0.61
c.Sex	-0.04	0.20	69.34	-0.20	0.84
c.Skill_class:VTL_condition-4	-0.61	0.18	1927.56	-3.45	0.00
c.Skill_class:VTL_condition+4	0.20	0.18	1927.70	1.13	0.26
c.Skill_class:z.f0_modulation	-0.03	0.97	1928.43	-0.03	0.98
VTL_condition-4:z.f0_modulation	-0.16	0.49	1928.50	-0.32	0.75
VTL_condition+4:z.f0_modulation	0.39	0.49	1928.32	0.79	0.43
c.Skill_class:c.Sex	0.01	0.40	69.04	0.02	0.98
VTL_condition-4:c.Sex	-0.19	0.18	1927.56	-1.08	0.28
VTL_condition+4:c.Sex	-0.58	0.18	1927.70	-3.30	0.00
z.f0_modulation:c.Sex	-0.25	0.97	1928.42	-0.26	0.80
c.Skill_class:VTL_condition-4:z.f0_modulation	0.00	0.98	1928.52	0.00	1.00
c.Skill_class:VTL_condition+4:z.f0_modulation	0.65	0.98	1928.35	0.66	0.51
c.Skill_class:VTL_condition-4:c.Sex	0.31	0.35	1927.56	0.88	0.38
c.Skill_class:VTL_condition+4:c.Sex	-0.64	0.35	1927.70	-1.83	0.07
c.Skill_class:z.f0_modulation:c.Sex	0.22	1.94	1928.43	0.11	0.91
VTL_condition-4:z.f0_modulation:c.Sex	0.34	0.98	1928.49	0.34	0.73
VTL_condition+4:z.f0_modulation:c.Sex	0.35	0.99	1928.32	0.35	0.73
c.Skill_class:VTL_condition-4:z.f0_modulation:c.Sex	-1.02	1.96	1928.52	-0.52	0.60
c.Skill_class:VTL_condition+4:z.f0_modulation:c.Sex	-0.75	1.97	1928.36	-0.38	0.70

Analysis of fourth formant (F4) modulations

Predictor	Estimate	Std. Error	df	t value	Pr(>|t|)
(Intercept)	0.01	0.10	51.81	0.07	0.94
c.Skill_class	0.03	0.20	51.62	0.14	0.89
VTL_condition-4	-0.75	0.08	1927.47	-8.95	0.00
VTL_condition+4	-0.05	0.08	1927.57	-0.57	0.57
z.f0_modulation	0.31	0.46	1928.10	0.67	0.51
c.Sex	-0.06	0.20	51.95	-0.27	0.79
c.Skill_class:VTL_condition-4	-0.72	0.17	1927.46	-4.30	0.00
c.Skill_class:VTL_condition+4	0.01	0.17	1927.57	0.04	0.97
c.Skill_class:z.f0_modulation	0.09	0.92	1928.11	0.09	0.92
VTL_condition-4:z.f0_modulation	-0.51	0.47	1928.16	-1.10	0.27
VTL_condition+4:z.f0_modulation	0.04	0.47	1928.03	0.09	0.93
c.Skill_class:c.Sex	-0.09	0.41	51.75	-0.23	0.82
VTL_condition-4:c.Sex	-0.12	0.17	1927.47	-0.72	0.47
VTL_condition+4:c.Sex	-0.54	0.17	1927.57	-3.24	0.00
z.f0_modulation:c.Sex	0.35	0.93	1928.10	0.37	0.71
c.Skill_class:VTL_condition-4:z.f0_modulation	-0.54	0.93	1928.18	-0.58	0.56
c.Skill_class:VTL_condition+4:z.f0_modulation	0.70	0.93	1928.05	0.75	0.45
c.Skill_class:VTL_condition-4:c.Sex	0.67	0.33	1927.46	2.01	0.04
c.Skill_class:VTL_condition+4:c.Sex	-0.35	0.33	1927.56	-1.04	0.30
c.Skill_class:z.f0_modulation:c.Sex	-0.49	1.84	1928.11	-0.26	0.79
VTL_condition-4:z.f0_modulation:c.Sex	-0.81	0.94	1928.15	-0.86	0.39
VTL_condition+4:z.f0_modulation:c.Sex	-0.16	0.94	1928.03	-0.17	0.87
c.Skill_class:VTL_condition-4:z.f0_modulation:c.Sex	-0.33	1.87	1928.18	-0.18	0.86
c.Skill_class:VTL_condition+4:z.f0_modulation:c.Sex	-0.13	1.87	1928.05	-0.07	0.95

Analysis of apparent vocal tract length (aVTL) modulations

Predictors	Estimate	Std. Error	df	t value	Pr(>|t|)
(Intercept)	0.00	0.08	59.88	-0.01	1.00
c.Skill_class	0.00	0.17	59.64	0.02	0.98
VTL_condition-4	0.79	0.07	1927.53	11.18	0.00
VTL_condition+4	-0.07	0.07	1927.65	-0.94	0.35
z.f0_modulation	-0.21	0.39	1928.27	-0.54	0.59
c.Sex	0.08	0.17	60.05	0.47	0.64
c.Skill_class:VTL_condition-4	0.70	0.14	1927.52	4.94	0.00
c.Skill_class:VTL_condition+4	-0.12	0.14	1927.64	-0.84	0.40
c.Skill_class:z.f0_modulation	-0.03	0.78	1928.28	-0.04	0.97
VTL_condition-4:z.f0_modulation	0.33	0.39	1928.33	0.83	0.41
VTL_condition+4:z.f0_modulation	-0.19	0.40	1928.18	-0.47	0.64
c.Skill_class:c.Sex	0.06	0.33	59.80	0.17	0.86
VTL_condition-4:c.Sex	0.17	0.14	1927.53	1.17	0.24
VTL_condition+4:c.Sex	0.40	0.14	1927.65	2.80	0.01
z.f0_modulation:c.Sex	-0.10	0.78	1928.27	-0.13	0.89
c.Skill_class:VTL_condition-4:z.f0_modulation	0.32	0.79	1928.36	0.41	0.68
c.Skill_class:VTL_condition+4:z.f0_modulation	-0.50	0.79	1928.21	-0.63	0.53
c.Skill_class:VTL_condition-4:c.Sex	-0.44	0.28	1927.52	-1.57	0.12
c.Skill_class:VTL_condition+4:c.Sex	0.55	0.28	1927.64	1.94	0.05
c.Skill_class:z.f0_modulation:c.Sex	0.42	1.56	1928.28	0.27	0.79
VTL_condition-4:z.f0_modulation:c.Sex	0.34	0.79	1928.33	0.43	0.67
VTL_condition+4:z.f0_modulation:c.Sex	-0.07	0.80	1928.19	-0.09	0.93
c.Skill_class:VTL_condition-4:z.f0_modulation:c.Sex	0.44	1.58	1928.36	0.28	0.78
c.Skill_class:VTL_condition+4:z.f0_modulation:c.Sex	-0.04	1.58	1928.21	-0.03	0.98

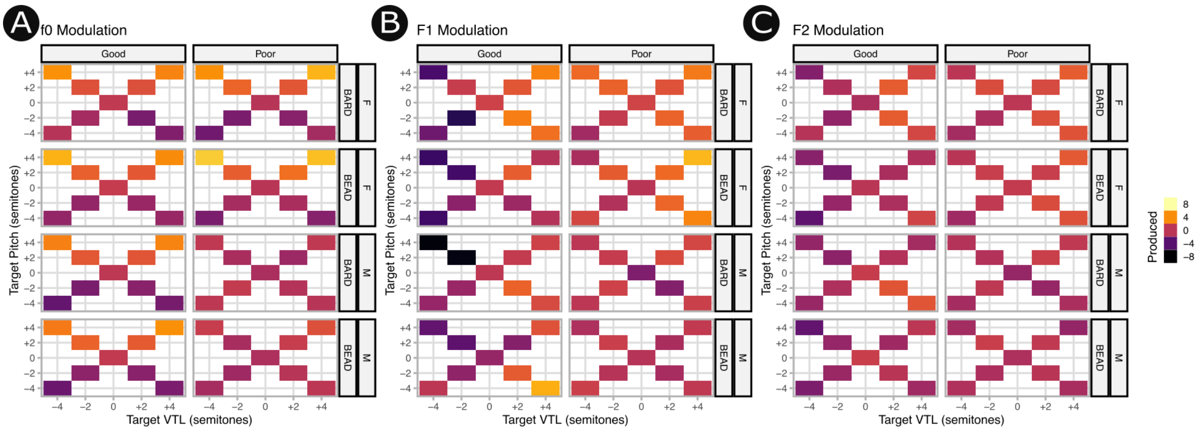

Figure SM3.1: Matrices depicting modulation of A) f0, B) F1, C) F2. Each panel depicts the matrix of conditions with vocal tract modulation on the x-axis and pitch modulation on the y-axis. The conditions on the diagonal form a continuum from masculinised to feminised voices. The off-diagonal contains less canonical combinations of vocal tract length and vocal pitch. The colour shading of each cell represents the estimates for each condition from linear mixed models (these models included an additional fixed effects factor for carrier word not included above). Cold colours indicate shifts towards lower frequencies and warm colours shifts towards higher frequencies.

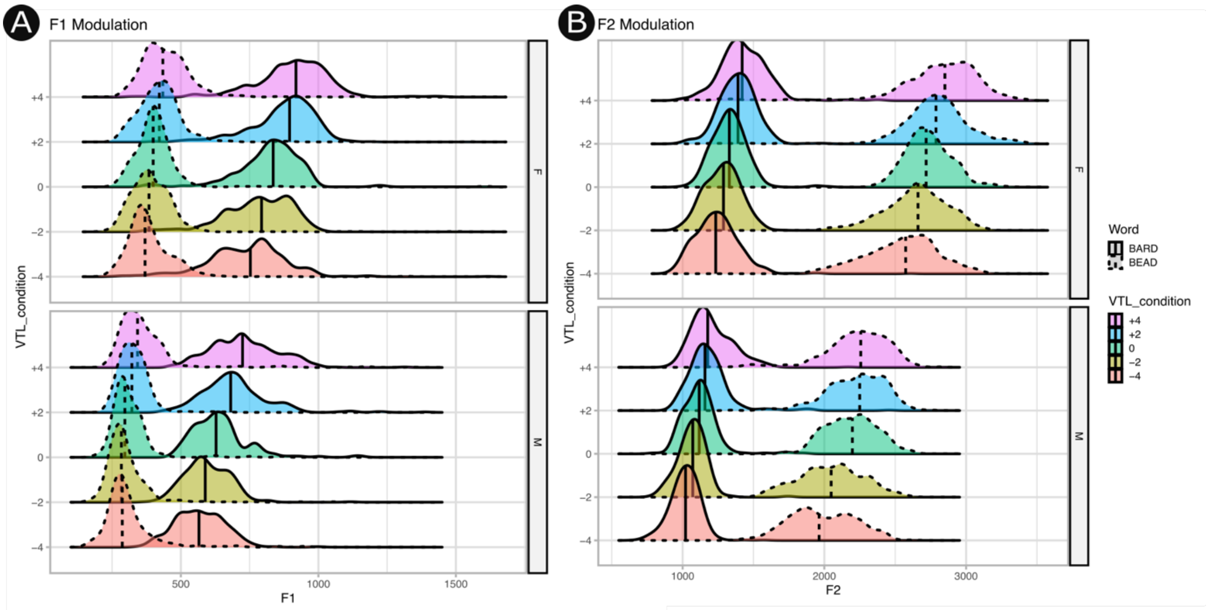

Figure SM3.2: Distributions of raw F1 and F2 values across vocal tract conditions and carrier words.

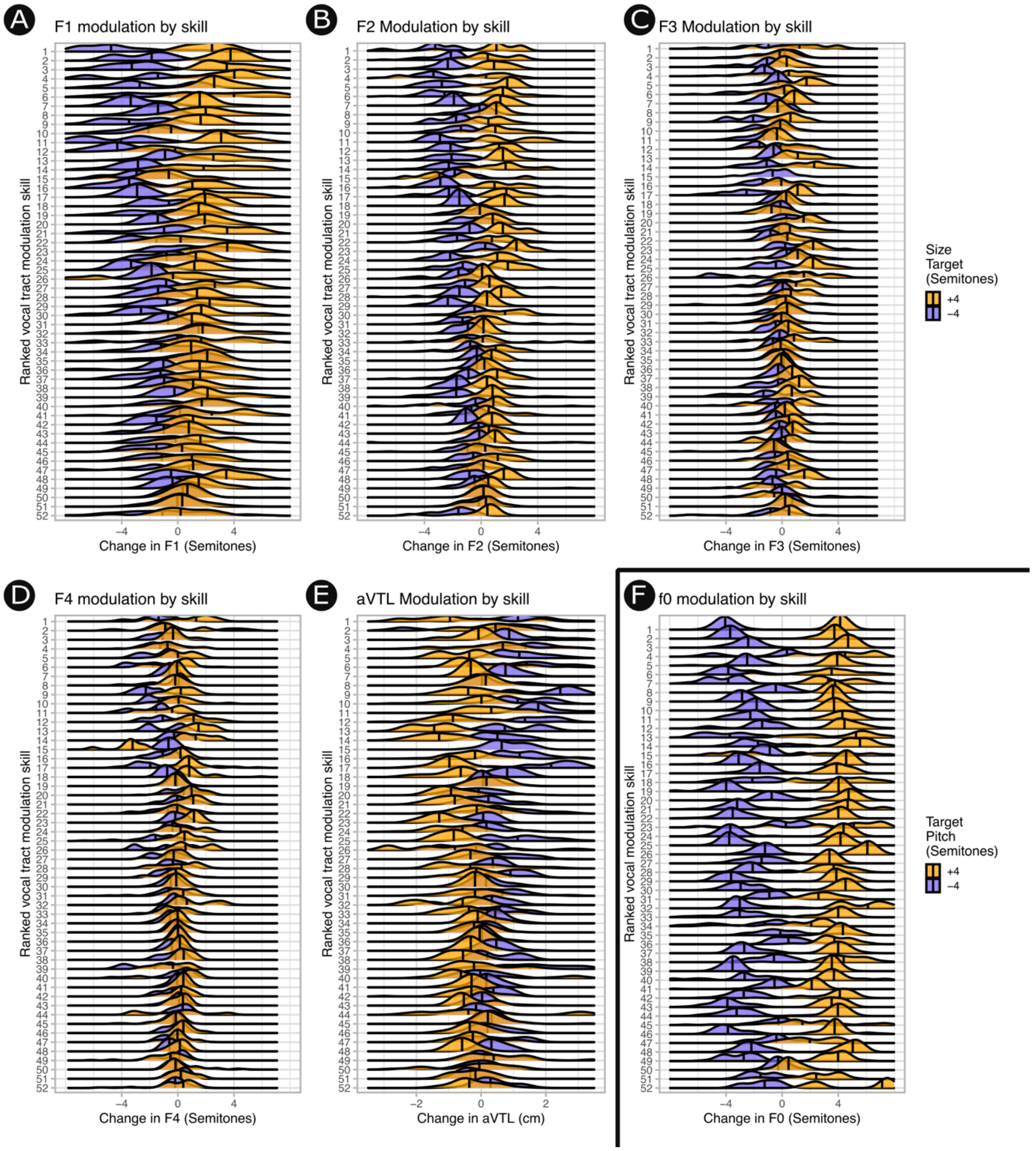

Figure SM3.3: Vocal modulation skill ridgelines. Modulation of acoustic measures (x-axis) sounding small (orange) or large (purple) for each participant (y-axis) for A) F1, B) F2, C) F3, D) F4, E) VTL. F) Equivalent figure for pitch modulation for imitating a high (orange) or low (purple) vocal pitch. Ridgelines for F1 and F2 demonstrate a strong relationship with vocal tract modulation skill. Ridgelines for F3 and F4 demonstrate that participants made minimal changes to these acoustic cues, though modulation is more prevalent with increasing vocal tract modulation rank. aVTL is a complex composite of F1-F4. Vocal pitch matching ability with strong across all participants irrespective of vocal tract modulation skill.
