# Supplementary material for: Individual differences in vocal size exaggeration
Source: Sci Rep. 2022 Feb 16;12:2611. doi: 10.1038/s41598-022-05170-6 (PMC8850436; doi:10.1038/s41598-022-05170-6)
Supplement: Supplementary file 3 — Supplementary Information 3. [file 41598_2022_5170_MOESM3_ESM.pdf]

Formant measurements using standard algorithms may be vulnerable to floor effects under certain conditions. In particular estimates of the first formant (F1) are unlikely to be observed if they are lower than the fundamental frequency (f0). While it is possible that this state may also reflect how humans perceive the voice, it may not reflect the bioacoustical mechanisms of the vocal tract. While F1 lower than f0 is not common it may occur in some circumstances in which f0 is particularly high (as in the voices of pre-pubescent children or female voices when vocal pitch is raised), and F1 is particularly low (certain phonemes that are encoded by low formants, sounding large).

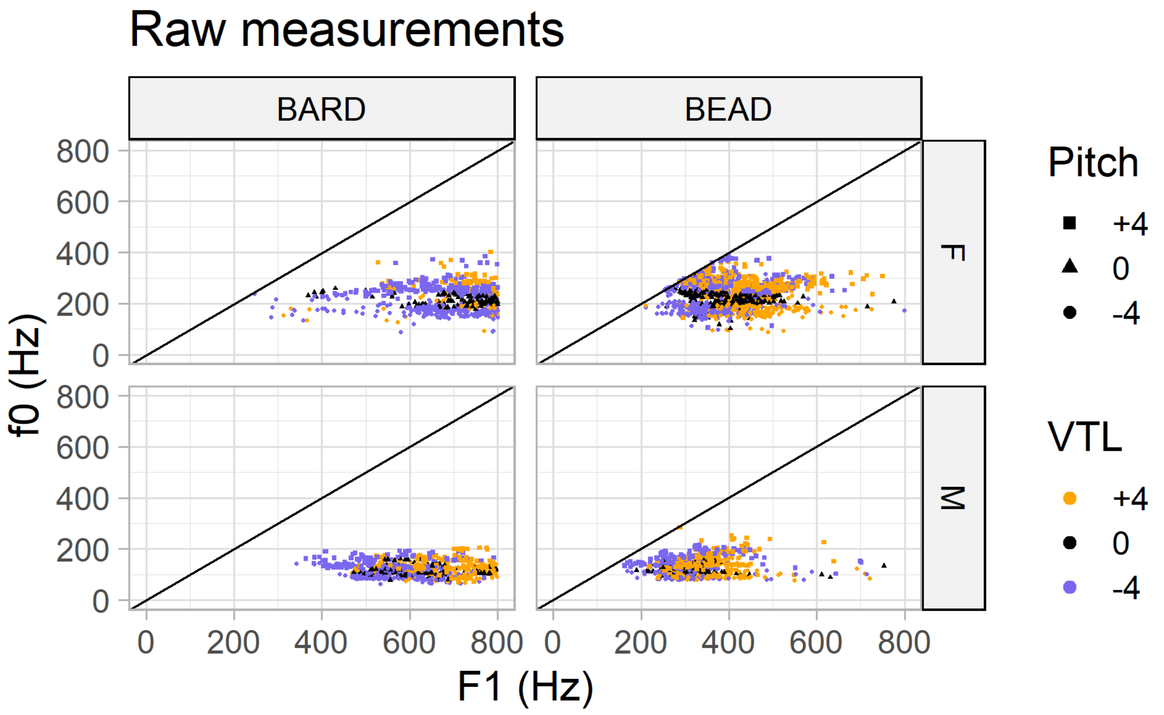


*Figure SM3.1: Scatterplots showing corresponding values of F1 and F0 measurements from individual audio recording. Separate panels are presented for the two carrier words (bard vs. bead) and speaker sexes (female vs male). The Linear-Predictive Coding (LPC) algorithm of Burg is purported to have a roving measurement floor, such that estimates of F1 are not considered if lower than f0. This floor is indicated by the black diagonal line (slope=1). The top right panel (female, BEAD) presents a case of potential floor induced measurement error. The highest instances of f0 when females spoke with a high pitch have f0 values in a range similar to F1 values in recordings where they used to carrier word BEAD to sound large (i.e., purple squares). It can be seen that the distribution of values abuts the diagonal measurement floor marker and it is likely that a small number of measurements may have had true F1 values lower than the estimates provided by LPC-Burg. Systematic measurement error is not predicted or evident in other conditions. X-axis range configured to highlight the slope=1 diagonal, some measurements may continue off plot.*

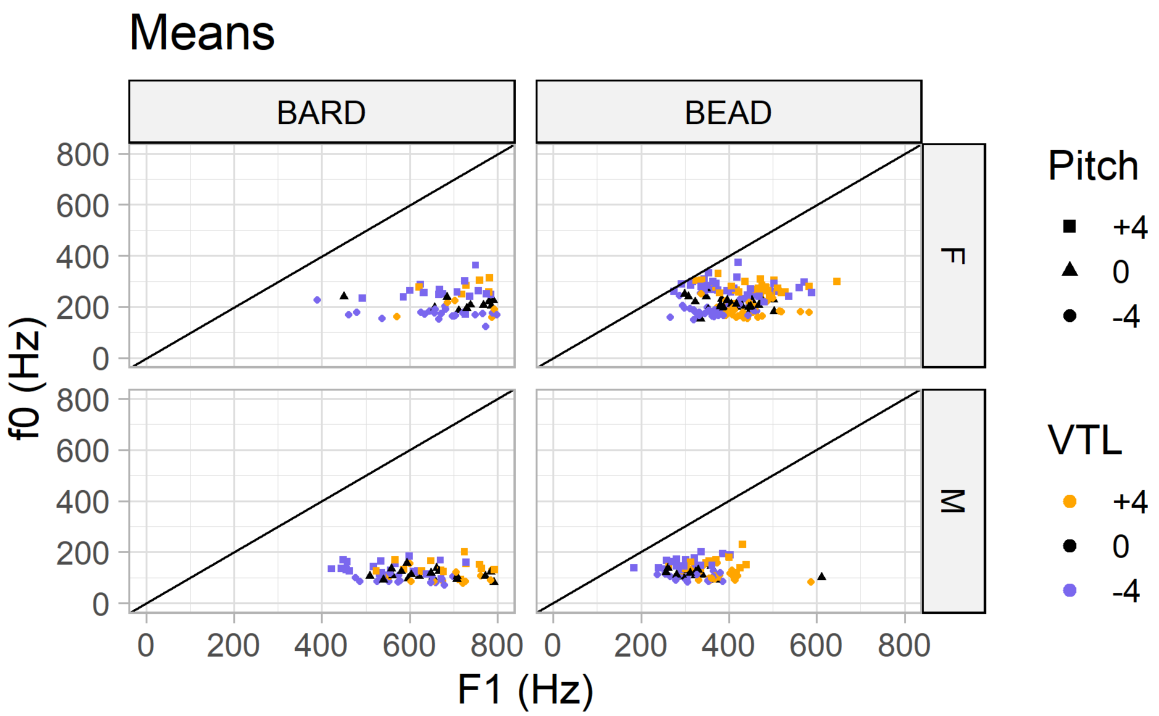


*Figure SM3.2: Within speaker means for each condition, sex, and carrier word. Systematic biases are not evident at the level of the speaker when females spoke the word bead with a high pitch sounding large. Notably the range of F1 values for females in the bead-large-high condition (top right, purple squares) was similar to the same speakers in the bead-large-low condition (top right, purple circles). This demonstrates that lowering the measurement floor (by lowering f0) did not produce on observable lowering of mean F1 values. Hence, while it is likely that LPC-Burg related measurement error had some influence on the data, this effect was liable to be small. X-axis range configured to highlight the slope=1 diagonal, some measurements may continue off plot.*
